# Effects of Changes in Osmolarity on the Biological Activity of Human Normal Nucleus Pulposus Mesenchymal Stem Cells

**DOI:** 10.1155/2022/1121064

**Published:** 2022-04-23

**Authors:** Hua Zhang, Wei Li, YaoHong Wu, Shengquan Zhang, Jie Li, Letian Han, Haoyu Chen, Ziyu Wang, Cailiang Shen, Yinshun Zhang, Hui Tao

**Affiliations:** ^1^Department of Orthopedics & Spine Surgery, The First Affiliated Hospital of Anhui Medical University, Hefei, 230022 Anhui, China; ^2^Department of Orthopedics, Huaibei People's Hospital, Huaibei, 235000 Anhui, China; ^3^Department of Spinal Surgery, Ganzhou People's Hospital, Ganzhou, 341000 Jiangxi, China; ^4^Department of Biochemistry and Molecular Biology, School of Basic Medical Sciences, Anhui Medical University, Hefei, 230022 Anhui, China; ^5^Department of Clinical Laboratory, The First Affiliated Hospital of Anhui Medical University, Hefei, 230022 Anhui, China

## Abstract

The expansion and maintenance of the NPMSC (nucleus pulposus mesenchymal stem cell) phenotype are considered as potential therapeutic tools for clinical applications in intervertebral disc tissue engineering and regenerative medicine. However, the harsh microenvironment within the intervertebral disc is the main limitation of its regeneration. The osmolarity of the intervertebral disc is higher than that of other tissues, which has an important influence on the biological characteristics of NPMSCs. In this study, we observed the effect of different osmolarities on the biological characteristics of human normal NPMSCs cultured in vitro and explored the role of osmolarity in intervertebral disc degeneration. Our data demonstrated that the change in osmotic pressure has an important effect on the biological activity of NPMSCs, and this effect may occur through the P16^INK4A^/Rb pathway. This study provides a theoretical basis for the future treatment of intervertebral disc degeneration.

## 1. Introduction

Low back pain is common, and its prevalence is increasing due to the aging of the population, seriously affecting the lives and health of patients. This condition is a major burden on society because of its high medical and financial costs [1, 2]. Studies have shown that intervertebral disc degeneration (IVDD) is an important pathological factor in back pain [3, 4]. IVDD is thought to be caused by a number of factors, including aging, biological factors, mechanical loading, smoking, and decreased cellular nutrition [5]. Degeneration of the intervertebral disc is characterized by damage to the nucleus pulposus (NP) cell function and reduced cell number, including degeneration of the annulus fibrosus, resulting in a disturbed ECM structure [6, 7]. The intervertebral disc connects two adjacent vertebral bodies, and no blood vessels directly supply nutrients. This structure is composed of three parts: annulus fibrosus, NP, and cartilage endplate [8]. The NP is located in the center of the intervertebral disc and has a unique biological environment, which is characterized by hyperosmolarity, nutrient deficiency, acidity, and hypoxia [9]. Normal intervertebral discs lack blood vessels and a nutrient supply, and metabolites are mainly eliminated through the diffusion of the cartilage endplate pathway [10] because in a hypoxic environment, cells primarily provide energy through glycolytic metabolism [11, 12]. As the degree of IVDD increases, the microenvironment of the cells in the intervertebral disc will change, the glucose supply will decrease, the oxygen supply will change, acidity will increase, and osmotic pressure will also change. These changes further aggravate IVDD and form a vicious cycle [13, 14].

Studies have shown that NP cell damage is an important cause of IVDD [15]. Aggrecan and collagen are the most important extracellular matrix in the nucleus pulposus: aggrecan provides the swelling pressure required to increase water content and the collagen provides resistance to swelling. The nucleus pulposus possesses mostly collagen II [16]. NP cells play an important role in maintaining the metabolic balance of the extracellular matrix. NP cell damage can cause disorder of the matrix metalloproteinase (MMP) secretion, leading to the degradation of the extracellular matrix, and the water content of nucleus pulposus will decrease, resulting in IVDD [17]. Therefore, current research on the treatment of IVDD is mainly focused on preventing the degradation of the extracellular matrix and increasing the number of NP cells [18]. Blanco et al. [19] identified nucleus pulposus mesenchymal stem cells (NPMSCs) in human degenerated NP tissues; these cells could replicate and undergo adipogenesis, osteogenesis, and chondrogenesis, indicating they are potential candidates for cell therapy in IVDD. Stimulating the proliferation and maintenance of the endogenous NPMSC phenotype may be a new strategy for the treatment of IVDD. However, due to the complex microenvironment of the intervertebral disc, regeneration and repair of NPMSCs are difficult.

The NP tissue of the intervertebral disc contains a high level of hydrophilic extracellular matrix, and the main component is proteoglycan [20]. Aggrecan increases intervertebral disc tissue permeability and resistance to pressure, and degradation of aggrecan can lead to damage to intervertebral disc function and the occurrence of IVDD. Aggrecan plays an important role in maintaining the hydration state and osmolarity of intervertebral discs [21]. Sulfated glycosaminoglycan is highly negatively charged and adsorbs cations and water molecules, thereby increasing the osmolarity of the tissue, promoting water entry in the intervertebral disc tissue, and then increasing the expansion pressure of the tissue to bear the compressive stress load of the spine [22, 23]. The various movements and loads of the spine throughout the day lead to dynamic changes in the osmolarity of the NP tissue. When the load stress exceeds the osmolarity of the NP tissue, water is squeezed out of the NP, and the osmolarity increases. When the stress is reduced, water is reabsorbed in the NP tissue to reduce osmolarity; thus, the osmolarity in the NP tissue is dynamically changing [24, 25].

Aging is one of the factors of nucleus pulposus cell damage. The P16^INK4a^/Rb signaling pathway is an important cellular senescence pathway and also an important pathway of cell proliferation. During normal cell proliferation, Cyclin D1 forms a complex with CDK4/6 to phosphorylate the Rb protein, and the phosphorylated Rb protein is dissociated from the transcription factor E2F, which promotes cell transition from G1 to S phase and enhances cell proliferation [26]. The P16^INK4A^ protein is an inhibitor of CDK4/6, which can compete with Cyclin D1 to bind CDK4/6, inhibit CDK4/6-mediated phosphorylation of Rb protein, prevent cells from entering the S phase from the G1 phase, and inhibit cell proliferation [26, 27]. Messmer et al. [28] found that an increase in tear osmotic pressure is the main cause of ocular inflammation and corneal stem cell apoptosis and aging. Many studies have shown that as cells age, the level of P16^INK4A^ increases [29–31].

Studies have determined that the osmolarity of NP tissue is 430–496 mOsm/kg H_2_O [32, 33], which is much higher than the normal osmotic pressure level of the human body. As an important factor for survival in the intervertebral disc tissue, osmolarity has a major effect on the activity of NPMSCs. Current research on the effect of osmotic pressure on NPMSCs mostly focuses on the intervertebral discs of cattle, mice, dogs, etc. In this study, for the first time, human normal NPMSCs were used to research the effect of changes in osmolarity on their biological activities and to explore the role of osmolarity in IVDD.

## 2. Materials and Methods

### 2.1. Cell Isolation and Culture

All experiments in this study were approved by the Ethics Committee of the First Affiliated Hospital of Anhui Medical University. In the experiment, intervertebral disc samples (*n* = 6, males = 3 and females = 3, aged 18-40 years, disc level T12-L3, and Grade I or II) were collected from patients undergoing lumbar fracture surgery at the First Affiliated Hospital of Anhui Medical University. According to the Pfirrmann disc degeneration grading system, the patients with disc degeneration were classified as Grade I or Grade II. We refer to relevant references to isolate and expand NPMSC cells [19, 34–36]. Under sterile conditions, the NP (nucleus pulposus) is separated from the AF (annulus fibrosus), making sure there is no any visible contamination by the AF or any other tissue. Then, scissors were used to cut the NP tissue into pieces, and the tissue was placed in a cell incubator and digested with 0.2 mg/ml collagenase II (Gibco, USA) for 4 to 6 hours. The cells were centrifuged, and the supernatant was discarded. The partially digested tissue, along with liberated cells, was cultured in the MSC expansion medium, which consisted of Dulbecco's modified Eagle's medium-low glucose (HyClone, USA), 10% fetal calf serum (Gibco, USA), and 1% penicillin/streptomycin (Gibco, USA) at 37°C in 5% CO_2_. After 24–48 hours, the cells began to adhere to the bottle and underwent the first fluid change, followed by a change of medium every 3 days. When the cell confluence reached approximately 90%, the cells were subcultured at 1 : 3, and the third generation of cells was selected for the experiments.

### 2.2. Immunophenotype of NPMSCs

After trypsin (Biosharp, USA) digestion of NPMSCs, the cells were washed with phosphate-buffered saline (PBS, Sigma, USA) and centrifuged to generate a 100 *μ*l cell suspension. CD105-PE, CD90-PE, CD73-PE, CD45-PE, CD34-PE, and HLA-DR-PE (eBioscience, USA) monoclonal antibodies were added to each tube. The samples were incubated in the dark for 30 min. After the cells were washed with PBS, they were resuspended in 400 *μ*l of PBS, and then, flow cytometry (BD, USA) was used to detect the percentage of positive cells and the fluorescence intensity. An isotype control (eBioscience, USA) was used for each tube.

### 2.3. Multilineage Differentiation

The isolated cells were plated in a 6-well plate and then cultured with mesenchymal stem cell differentiation medium (Cyagen Biosciences, Guangzhou, China), including osteogenic differentiation, adipogenic differentiation, and chondrogenic differentiation media. The cells were cultured for 21 days for osteogenic differentiation, 28 days for adipogenic differentiation, and 28 days for chondrogenic differentiation. Then, the culture medium was discarded, and the cells were stained with Alizarin Red, Oil Red O, and Alcian Blue. Finally, the staining results were observed under an inverted microscope.

### 2.4. Preparation of Four Experimental Groups

Cells isolated from the intervertebral disc tissue were cultured and passaged. Sterilized NaCl and deionized water were added to the medium to prepare the culture medium with different osmotic pressure levels, and the osmotic pressure was monitored using a freezing point osmotic pressure instrument (Fiske, USA). Media with four osmotic pressure levels, namely, 250 mOsm/kg H_2_O (group 1), 300 mOsm/kg H_2_O (group 2), 450 mOsm/kg H_2_O (group 3), and 600 mOsm/kg H_2_O (group 4), were used. Only the osmotic pressure was different in each group; the other culture conditions were the same.

### 2.5. Cell Proliferation Assay

Cell Counting Kit-8 (CCK-8) assays were used to detect the proliferation of the NPMSCs cultured under different osmotic pressure conditions. NPMSCs were seeded in 96-well plates at a density of 5 × 10^3^ cells/ml. These cells were cultured for 1, 3, 5, 7, 9, and 11 days in the four groups (1, 2, 3, and 4). The cells were incubated with 10 *μ*l of CCK-8 reagent (Dojindo, Japan) at 37°C in the dark for 2.5 hours. No cells were added to the isotype group. A SpectraMax microplate reader was used to measure the absorbance of different groups at 450 nm.

### 2.6. Apoptosis Assay

The isolated cells were planted in a 6-well plate. These cells were cultured in four groups (1, 2, 3, and 4) for 5 days in different osmotic media. The cells were digested with trypsin without EDTA (Biosharp, USA) and washed twice with PBS. The harvested cells were incubated with 5 *μ*l of Annexin V-FITC and 5 *μ*l of propidium iodide (PI; KeyGEN BioTECH, China) for 5 min at 37°C in the dark. Flow cytometry (BD, USA) was used to detect the percentage of apoptotic cells.

### 2.7. Cell Cycle Analysis

PI (Beyotime, China) staining was used to analyze the cell cycle. Briefly, after NP cells were treated with medium with different osmolarities for 5 days, they were digested with 0.25% trypsin and fixed with cold ethanol overnight. The cells were washed with PBS to remove ethanol. The prepared 500 *μ*l PI/RNase A staining working solution was added to the tube and incubated for 30 min in the dark at room temperature. After the cells were washed with PBS, the fractions of each phase of the cell cycle were detected by flow cytometry.

### 2.8. SA-*β*-Gal Staining Assay

The cells were seeded in a 6-well plate and then cultured in the above four different osmotic pressure groups for 5 days. SA-*β*-Gal staining was performed according to the manufacturer's instructions (Beyotime, China). Then, the staining results were observed under an inverted microscope. Senescent cells were stained. The percentage of positive cells to the total number of cells was determined.

### 2.9. Real-Time PCR Analysis

The cells were cultured for 7 days with the above four types of media with different osmotic pressures. Then, a TRIzol reagent was used to extract total RNA. Total RNA was reverse transcribed into cDNA using a reverse transcription reagent (TaKaRa, Japan). GAPDH was used as an internal reference gene. A SYBR Premix Ex Taq PCR kit (TaKaRa, Japan) and LightCycler system (Roche, Switzerland) were used to analyze the obtained cDNA for real-time quantitative PCR (RT-qPCR). The primers used for RT-qPCR are listed in Table 1 and were synthesized by Sangon Biotech (Shanghai, China). The mRNA expression of target genes was calculated with the 2^−△△Ct^ method.

### 2.10. Western Blot Analysis

After 7 days of culture with media with different osmotic pressures, the four groups of cells were treated with a RIPA lysis buffer (Beyotime, China), and total protein was extracted. The extracted protein samples from each group were separated by SDS-PAGE and transferred to PVDF membranes. The PVDF membranes were incubated with primary antibodies (*β*-actin: Bioworld, BS6007M; Aggrecan: Abcam, ab3778; Collagen II: Abcam, ab188570; P16^INK4A^: Abcam, ab108349; Rb: Abcam, ab181616) and horseradish peroxidase- (HRP-) conjugated secondary antibodies (Beyotime, China). After the protein bands were visualized using an ECL Plus system (Thermo, USA), their gray values were measured by Quantity One software (Bio-Rad, USA). The protein expression of target molecules was normalized to that of *β*-actin.

### 2.11. P16^INK4A^ Knockdown by Small-Interfering RNA (siRNA)

The siRNA was designed and provided by GenePharma; we assessed three sequences to identify the most effective siRNA. The sequences are as follows: P16^INK4A^-527 (5′-3′ CGGGAAACUUAGAUCAUCATT, UGAUGAUCUAAGUUUCCCGTT), P16^INK4A^-927 (5′-3′ GCAGAACCAAAGCUCAAAUTT, AUUUGAGCUUUGGUUCUGCTT), and P16^INK4A^-676 (5′-3′ CCGUAAAUGUCCAUUUAUATT, UAUAAAUGGACAUUUACGGTT). First, we screened for the most effective sequence, and we grew cells in 6 wells. When the cell confluence reached approximately 80%, according to the kit instructions for P16^INK4A^ siRNA transfection, we cultured the cells in a 37°C incubator and replaced the complete medium after 6 hours. Forty-eight hours later, we assessed the P16^INK4A^ mRNA expression, and after 72 hours, the P16^INK4A^ protein expression was determined. The most effective sequence was selected for subsequent tests. After selecting the most effective sequence, we transfected it according to the instructions. Then, we incubated the samples in a 37°C cell incubator for 6 hours and replaced the medium with 4 types of medium with different osmotic pressure levels. The mRNA expression of related genes was detected after 48 hours, and the expression of target proteins was detected after 72 hours.

### 2.12. Statistical Analysis

All data in this study are expressed as the mean ± standard error of three independent replicates. The significant difference between groups was analyzed by one-way analysis of variance (ANOVA) using SPSS 19.0 software. A *P* value < 0.05 was considered statistically significant.

## 3. Results

### 3.1. Isolation and Characterization of NPMSCs

**Figure 1:**
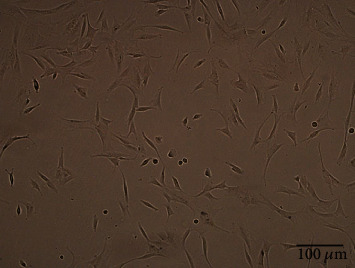
The cultured cells of the third generation were fusiform and adherent.

**Figure 2:**
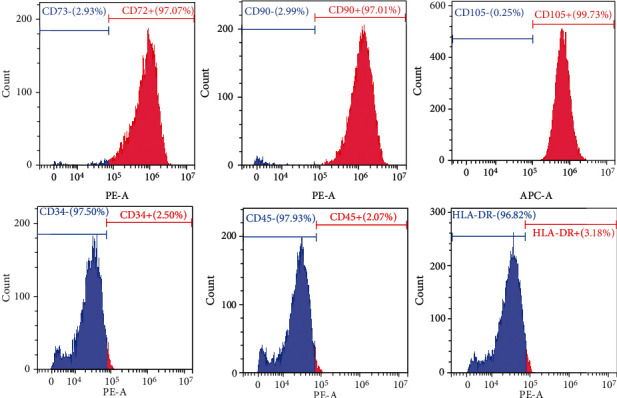
The cells were highly positive for CD73, CD90, and CD105 and were negative for CD34, CD45, and HLA-DR.

**Figure 3:**
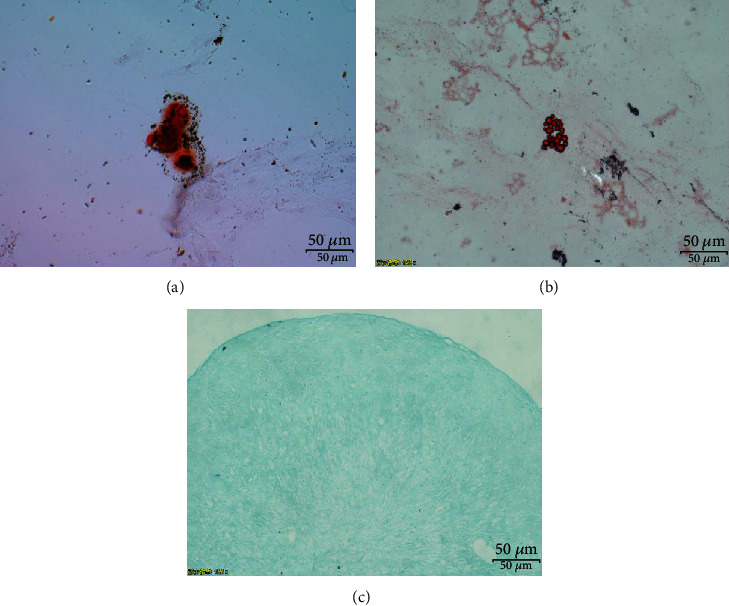
(a) Alizarin Red staining showed that cells formed mineralized nodules. (b) Oil Red O staining revealed that cells produced intracellular lipid vacuoles. (c) Alcian Blue staining indicated that cells exhibited sulfated proteoglycan.

After the primary cells were cultured for 4 to 5 days, the isolated and cultured cells adhered to the bottle. Under a microscope, the cells adhered to the bottle and had a spindle shape. The cells became confluent in approximately 20 days, and the speed of confluence increased after passaging. The cell morphology of the P2 and P3 generations remained uniform, and the cells had a spindle shape (Figure 1). Flow cytometric analysis showed that the cells had high expression of CD73, CD90, and CD105 and low expression of CD34, CD45, and HLA-DR (Figure 2). After 21 days of osteogenic differentiation, mineralized nodules could be detected by Alizarin Red staining. After 28 days of adipogenic differentiation, Oil Red O staining showed that lipid vesicles were produced in the cells. After 28 days of chondrogenic differentiation, Alcian Blue staining showed the presence of sulfated proteoglycan in the cells (Figure 3).

### 3.2. NPMSCs Cultured under Different Osmotic Pressures

**Figure 4:**
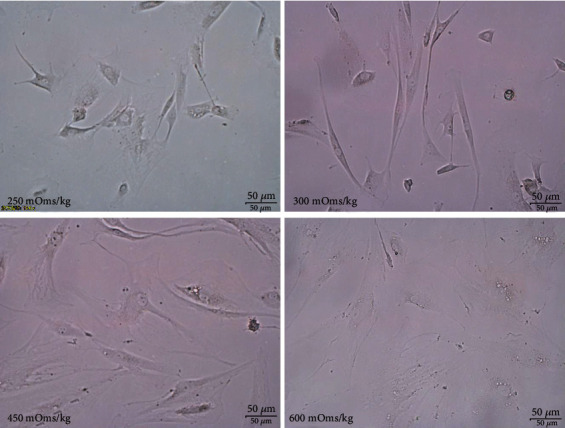
Compared with the isotonic pressure group, the cells in the hypertonic and hypotonic groups became larger, flatter, and irregular in shape.

We cultured NPMSCs in four groups under different osmotic pressures. After five days of culture, morphological changes in the cells were observed, and their morphology was recorded by photographs. As the osmotic pressure changed, the cell morphology also changed. Compared with the isoosmotic pressure group, the cells in the hypertonic group became larger, flatter, and irregular in shape. The morphology of the cells in the hypotonic group also showed the same change. (Figure 4).

### 3.3. Cell Proliferation of NPMSCs

**Figure 5:**
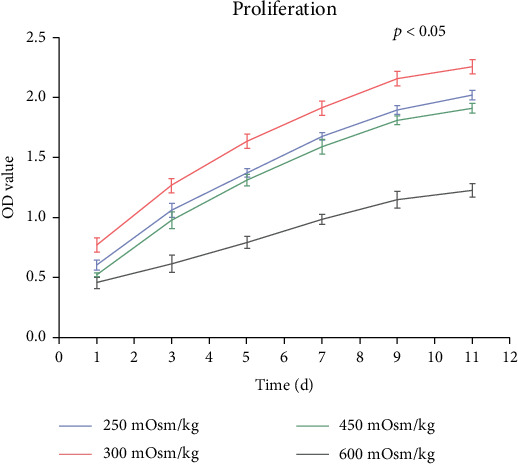
The proliferation rate of mesenchymal stem cells in nucleus pulposus was the highest under normal osmolarity, while cell proliferation was inhibited under hypoosmotic and hyperosmotic conditions.

After the cells in the four groups (1, 2, 3, and 4) were grown for 11 days, the proliferation rate of group 1 was lower than that of group 2, and the cell proliferation rates of groups 3 and 4 decreased and were lower than that of group 2. The cell proliferation rate of group 4 was lower than that of group 3 (Figure 5).

### 3.4. Apoptosis Assay of NPMSCs

**Figure 6:**
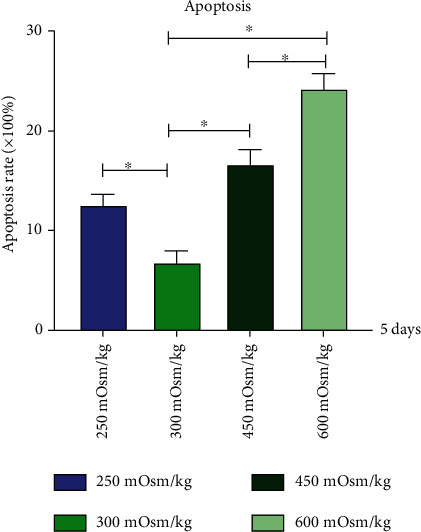
Apoptosis rate of cells cultured at osmotic pressure of four groups. Each value is the mean ± SEM; ^∗^*P* < 0.05.

The cells were cultured in four groups, 1, 2, 3, and 4, for 5 days. The apoptosis rate of group 2 was the lowest. The apoptotic rates of groups 1, 3, and 4 were significantly higher than that of group 2. The apoptosis rate of group 4 was higher than that of group 3 (Figure 6).

### 3.5. The Cell Cycle of NPMSCs

**Figure 7:**
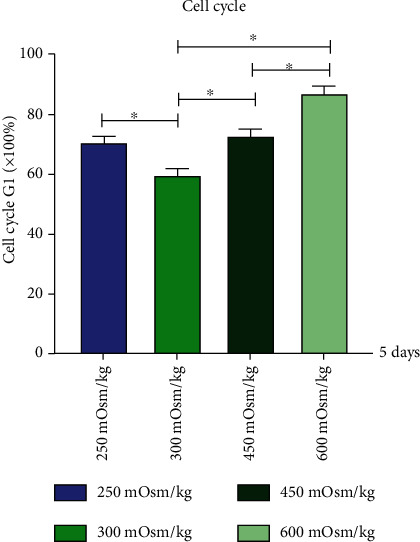
Percentage of G1 phase of cell cycle cultured at osmotic pressure of the four groups. Each value is the mean ± SEM; ^∗^*P* < 0.05.

We cultured the above 4 groups of cells for 5 days. The results showed that compared with the isotonic pressure group, the hypotonic group and the hypertonic group showed inhibition of the cell cycle, and the higher the osmotic pressure, the more obvious the inhibitory effect on the cells was (Figure 7).

### 3.6. SA-*β*-Gal Activity of NPMSCs

**Figure 8:**
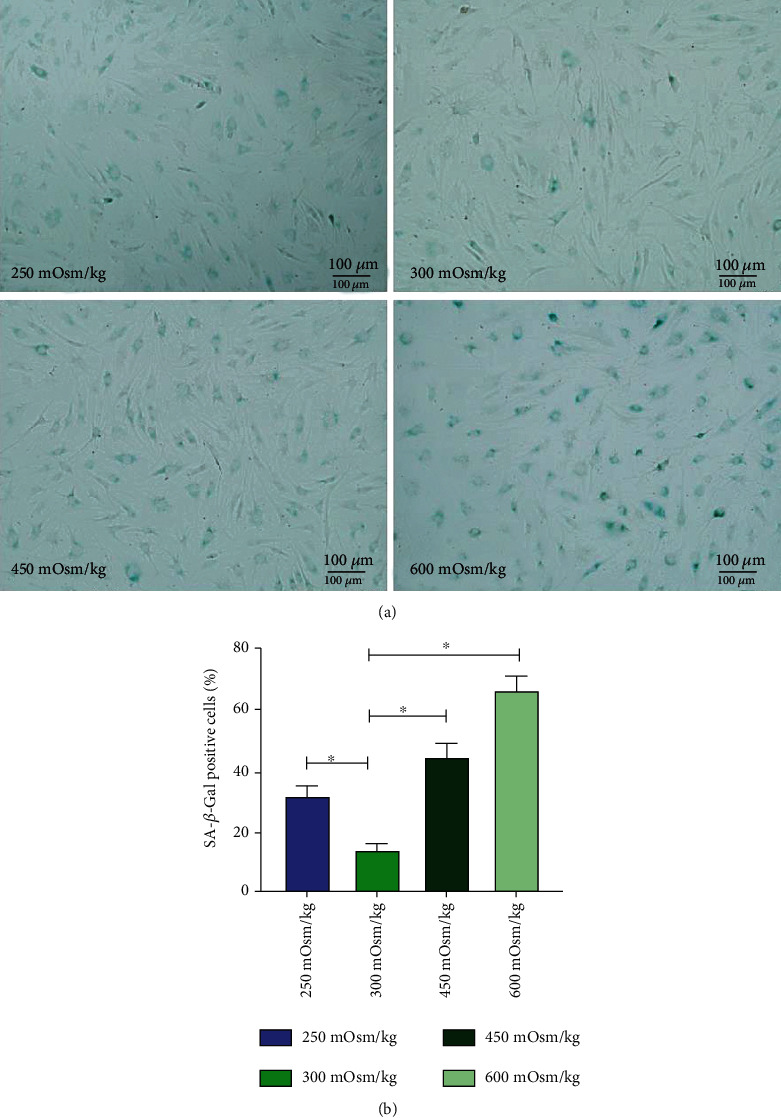
Four groups of cell senescence staining cultured at osmotic pressure concentration. Each value is the mean ± SEM; ^∗^*P* < 0.05.

The SA-*β*-Gal activity of the hypotonic group and the hypertonic group was significantly higher than that of the isotonic group. Both hypotonic and hypertonic conditions can promote cell senescence. The greater the osmotic pressure, the more significant the enhancement of cell senescence was (Figure 8).

### 3.7. Gene Expression of NPMSCs

**Figure 9:**
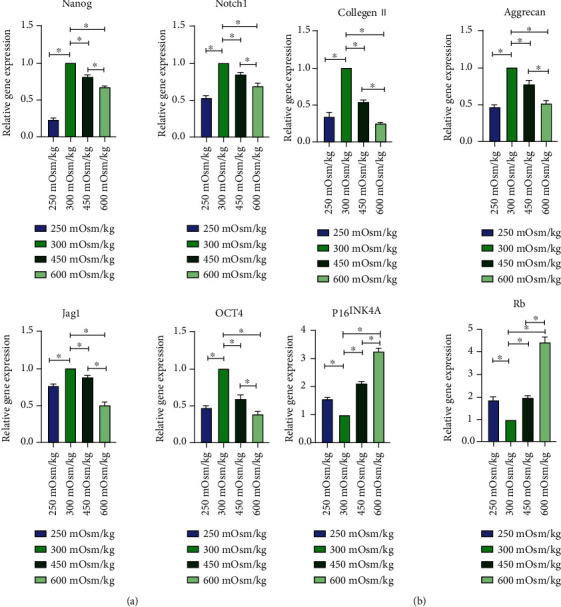
(a, b) The expression levels of related genes in four groups cultured under osmotic pressure. Each value is the mean ± SEM; ^∗^*P* < 0.05.

NPMSCs were cultured under four different osmotic pressures for 7 days. Compared with that in group 2, the expression of stemness genes (Nanog, Notch1, Jag1, and OCT4) and functional genes (Collagen II and Aggrecan) was downregulated in group 1, group 3, and group 4, and the expression in group 4 showed greater downregulation than that in group 3. The expression of the P16^INK4A^ and Rb genes was also upregulated, and group 4 showed greater upregulation than group 3 (Figure 9).

### 3.8. Protein Expression of NPMSCs

**Figure 10:**
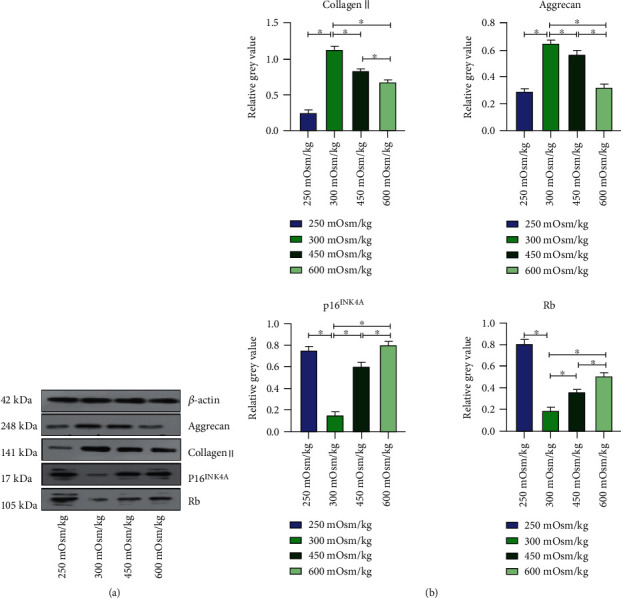
(a, b) Expression levels of target proteins in cells cultured under osmotic pressure of the four groups. Each value is the mean ± SEM; ^∗^*P* < 0.05.

NPMSCs were cultured in groups 1, 2, 3, and 4 for 7 days, and Western blotting results showed that compared with that in group 2, the expression of Collagen II and Aggrecan in group 1, group 3, and group 4 was downregulated, and group 4 showed greater downregulation than group 3. The expression of P16^INK4A^ and Rb was also upregulated, and group 4 showed greater upregulation than group 3 (Figure 10).

### 3.9. The Effect of P16^INK4A^ Knockdown on the NPMSC Activity

**Figure 11:**
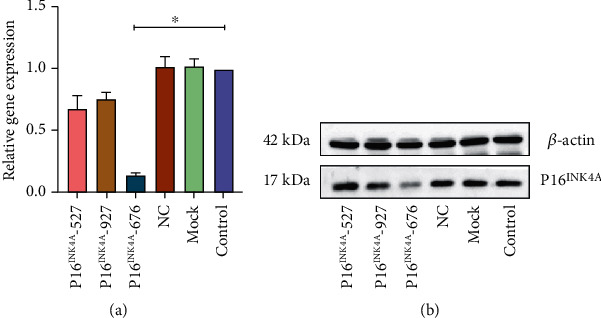
The results of three-sequence screening; PCR (a) and WB (b) showed that P16^INK4A^-676 was effective for transfection. Each value is the mean ± SEM; ^∗^*P* < 0.05.

After knockdown of P16^INK4A^ by siRNA transfection, the effects were verified by PCR and Western blots, which showed that the expression of P16^INK4A^ was significantly lower than that of the control group (Figure 11).

**Figure 12:**
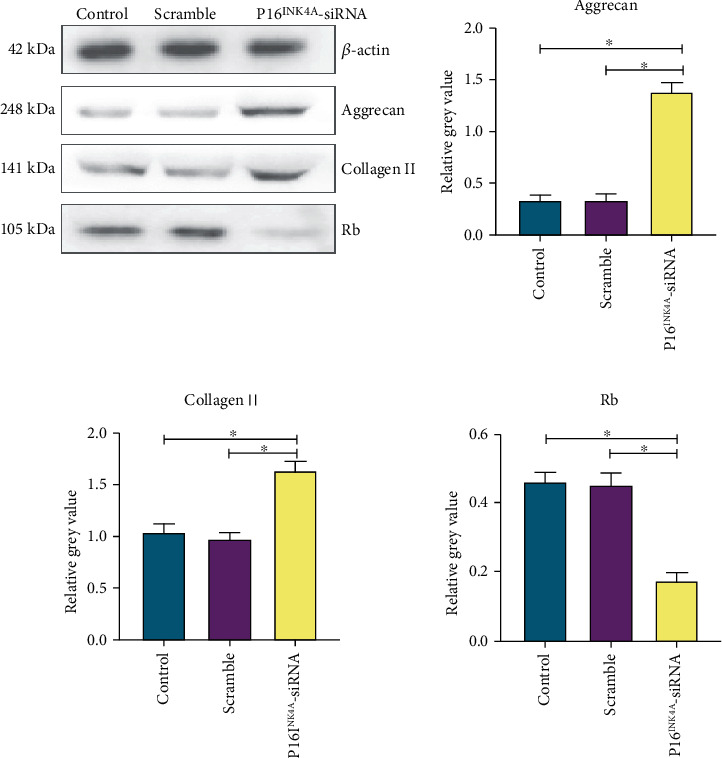
Selected the osmotic pressure of 600 mOsm/kg H_2_O for negative control analysis. Each value is the mean ± SEM; ^∗^*P* < 0.05.

**Figure 13:**
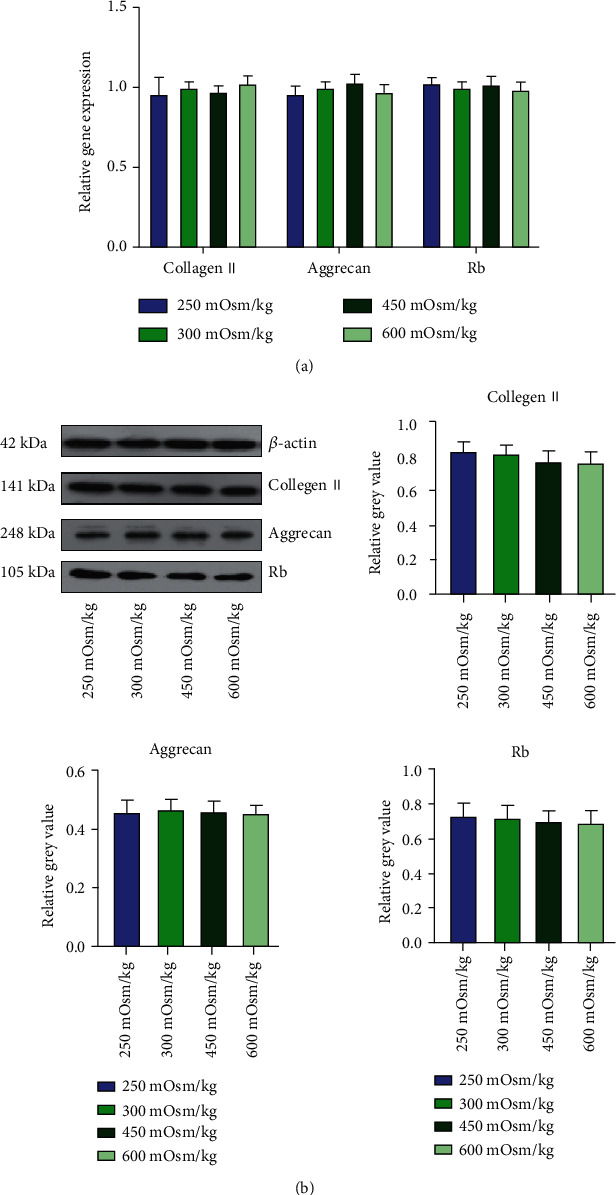
(a) Knockdown of P16^INK4A^ by siRNA transfection and detection of the mRNA expression of related genes after culturing cells with different osmotic pressures for 48 hours. (b) Knockdown of P16^INK4A^ by siRNA transfection; after culturing cells with different osmotic pressures for 72 hours, the expression of the target protein was detected. Each value is the mean ± SEM; ^∗^*P* < 0.05.

In this study, we found that group 4 had the greatest effect on the biological activity of the NPMSCs, and then, we selected the osmotic pressure of 600 mOsm/kg H_2_O for negative control analysis (Figure 12). After knocking down P16^INK4A^, we continued to culture the cells under the above 4 osmotic pressure conditions and then tested them. The mRNA and protein levels of Collagen II, Aggrecan, and Rb were detected, and the results showed no significant differences among the groups (Figure 13).

## 4. Discussion

The intervertebral disc is a tissue with a special structure. Its biological environment is relatively closed, and blood vessels that directly supply nutrients are lacking, which results in this specific structure. As the degree of IVDD increases, the microenvironment of the intervertebral disc changes, and the osmotic pressure also changes [14]. Moreover, various movements and loads of the spine lead to changes in the osmotic pressure in the NP tissue. A study by Urban [37] showed that the osmotic pressure of NP tissue is between 450 and 550 mOsm/kg H_2_O in a day. However, the exact mechanism of the effect of osmotic pressure changes on NPMSCs is still unclear. This study simulated different osmotic pressure environments to culture human normal NPMSCs from intervertebral discs to study the effect of osmotic pressure on the biological activity of these cells.

Risbud et al. [34], Blanco et al. [19], and Tekari et al. [35] isolated MSC-like progenitor cells from nucleus pulposus by enzymatic digestion, and they fulfill nearly all morphological, immunophenotypic, and differentiation criteria described by the International Association for Stem Cell Therapy. The NP tissue selected in this study was derived from patients with lumbar vertebral fractures and was from normal intervertebral discs (Pfirrmann I or II). We isolated and cultured cells in vitro and found that the cells adhered to the bottle and grew in a radial spiral shape, with high expression of CD73, CD90, and CD105; low expression of HLA-DR, CD34, and CD45; and differentiation towards osteogenic, adipogenic, and chondrogenic lineages. We isolated cells in the same way as Risbud et al., Blanco et al., and Tekari et al., according to the evaluation criteria of the International Association for Stem Cell Therapy (ISCT) [38]; the isolated and cultured cells were NPMSCs.

Ishihara et al. [39] cultivated NP tissue in vitro and found that increased osmotic pressure could enhance the synthesis of proteoglycans, and the increase in proteoglycan synthesis was related to the osmotic pressure of the culture medium but unrelated to Na^+^ or Cl^−^. Neidlinger-Wilke et al. [40] increased the osmotic pressure of the medium in bovine NP cells from 300 mOsm/kg H_2_O to 500 mOsm/kg H_2_O, and the proteoglycan expression increased. Spillekom et al. [41] found that the synthesis of proteoglycan in dog NP cells was optimal when the osmotic pressure in the culture medium was 400 mOsm/kg H_2_O. Studies have also found that high osmotic pressure can inhibit the proliferation of NP cells and the synthesis of extracellular matrix and promote the apoptosis of NPMSCs [42–44]. The above findings on osmotic pressure on the biological activity of NP cells are different, which may be caused by the method of cell culture, the difference in cell species, and the different exposure conditions. In this study, we simulated different osmotic pressure environments in vitro and cultured NPMSC cells under different osmotic pressure conditions. Experimental results showed that compared with normal osmotic pressure, hypotonic and hypertonic environments inhibited the proliferation of NPMSCs. The higher the osmotic pressure, the more obvious the inhibition of NPMSC proliferation was.

High osmotic pressure disrupts the cell cycle, can arrest cells in the G1 phase, and inhibits the cell cycle [45–47]. These findings are similar to our research results. We used flow cytometry to detect the cell cycle of the NPMSCs cultured under different osmotic pressures and found that compared with the normal osmotic pressure group, the high osmotic pressure group showed arrest of cells in the G1 phase. The higher the osmotic pressure, the stronger the inhibitory effect is. Research shows that not only high osmotic pressure but also low osmotic pressure can impair the cell cycle progression of NPMSCs. Changes in osmotic pressure also affect the expression of extracellular matrix [45–47]. The results showed that when the osmotic pressure was decreased or increased, the expression of related genes (Nanog, Nocth1, Jag1, and OCT4) in NPMSCs decreased and the expression of Collagen II and Aggrecan was also reduced compared to that in the normal osmotic pressure group. These results suggested that abnormal changes in osmotic pressure will cause dysfunction of NPMSCs, which will lead to IVDD.

The P16^INK4A^/Rb signaling pathway plays an important role in cell senescence, and P16^INK4A^ can cause permanent cell growth arrest and cell senescence [48]. This study also confirmed these findings. We observed the morphological changes of cells cultured in vitro under different osmotic pressures by microscope and found that the cells in the hypotonic and hypertonic groups became larger and flatter, and the cell bodies were irregular in shape, which was consistent with the morphological changes of senescent cells [49, 50]. Research shows that compared with the normal osmotic pressure group, the hypotonic and hypertonic groups showed an increased proportion of cell senescence, as demonstrated by galactosidase staining; the expression of the P16^INK4A^ and Rb genes increased; and the expression of Collagen II and Aggrecan decreased. These results suggested that low and high osmotic pressures induced the upregulation of P16^INK4A^ gene expression in NPMSCs, causing damage to NPMSCs.

To further clarify the role of P16^INK4A^ and osmotic pressure in NPMSCs, we used siRNA transfection to knock down the expression of P16^INK4A^. We detected the P16^INK4A^ protein level by Western blots and the P16^INK4A^ mRNA level by PCR. We found that the expression of P16^INK4A^ in the siRNA-transfected cells was significantly lower than that in the control group. Both methods showed good transfection effects and high efficiency. After knockdown of P16^INK4A^, we found that there was no significant difference in the expression of the Collagen II, Aggrecan, and Rb genes after the osmotic pressure was changed, as shown by PCR and Western blots. Silencing of the P16^INK4A^ gene blocked the adverse effects of osmotic pressure on the expression of the Collagen II, Aggrecan, and Rb genes in NPMSCs.

This study confirmed that compared with the normal osmotic pressure group, the low osmotic pressure and high osmotic pressure groups showed activation of the P16^INK4A^/Rb signaling pathway. The expression of the P16^INK4A^ gene was upregulated, and the phosphorylation level of the Rb protein was reduced. Rb combined with the transcription factor E2F and prevented cells from entering the S phase from the G1 phase, and the cell cycle is inhibited. After knockdown of the P16^INK4A^ expression by siRNA, the P16^INK4A^/Rb signaling pathway was blocked, the change in osmotic pressure could not activate the P16^INK4A^/Rb signaling pathway, the expression of the Rb gene was not affected, and the cell cycle progression of NPMSCs was not blocked. Cell aging was inhibited, and the expression of extracellular matrix components did not decrease. Because of the time limit of siRNA transfection, we did not conduct long-term cell culture on the knockdown cells and did not perform more in-depth research on cell differentiation and proliferation.

We used the fetal bovine serum instead of human serum to culture the human NPMSCs. Differences in serum species may affect the test results, but human serum samples are limited. Second, the study was conducted in a 2D culture medium, and the response of NPMSCs in a 3D culture system may be different. Third, the research on the silencing of the P16^INK4A^ gene is mainly carried out in monolayer-cultured NPMSCs. Dedifferentiation and short-term effects are inevitable. Although these effects were biologically important, they may not be relevant to in vivo situations. The long-term effect in the body is very important. Although the exact mechanism of P16^INK4A^ has yet to be determined, knocking down P16^INK4A^ in NP cells may have some beneficial biological functions.

## 5. Conclusion

In summary, the results showed that changes in osmotic pressure have an important effect on the biological activity of NPMSCs; hypotonic or hypertonic can induce cell apoptosis and promote cell senescence. Studies have shown that this effect may occur through the P16^INK4A^/Rb pathway and affect the regeneration and restore functions of NPMSCs. By knocking down the P16^INK4A^ gene, the adverse effects of changes in osmotic pressure on cells can be reversed. This study provides a useful theoretical basis for the treatment of IVDD in the future.

## Figures and Tables

**Table 1:** Primers of target genes.

Gene	Forward (5′-3′)	Reverse (5′-3′)
GAPDH	GGAGCGAGATCCCTCCAAAAT	GGCTGTTGTCATACTTCTCATGG
Nanog	GCTTTGAAGCATCCGACTGT	TTTGGGACTGGTGGAAGAAT
Notch1	GCCTCAACATCCCCTACAAG	CACGAAGAACAGAAGCACAAA
Jag1	GGGTCACTGTCAGAATGAAATC	AGTCACTGGCACGGTTGTAG
OCT4	ACACTGCAGCAGATCAGCCAC	CCAGAGGAAAGGACACTGGTC
Aggrecan	GTGCCTATCAGGACAAGGTCT	GATGCCTTTCACCACGACTTC
Collagen II	CTTCCTACGGGGAATCTGTGT	CAATGGCGTTTTGGGTGTTC
P16^INK4A^	GGGTTTTCGTGGTTCACATCC	CTAGACGCTGGCTCCTCAGTA
Rb	TTGGATCACAGCGATACAAACTT	AGCGCACGCCAATAAAGACAT

## Data Availability

Data are available on request.
